# Evaluation and investigation of the cardiotoxicity of the potential anti-cholangiocarcinoma drug lanatoside C

**DOI:** 10.3389/ftox.2026.1783444

**Published:** 2026-05-25

**Authors:** Yu-Hai Ma, Chong-Fei Huang, Hai-Dong Ma, Jin-Yu Zhao, Wen-An Wang, Zi-He Dong, Yi Shao, Long Gao, Jian-Jun Chen, Wen-Bo Meng

**Affiliations:** 1 The First Clinical Medical College, Lanzhou University, Lanzhou, Gansu, China; 2 Department of Emergency, The First Hospital of Lanzhou University, Lanzhou, Gansu, China; 3 Gansu Province Key Laboratory Biotherapy and Regenerative Medicine, Lanzhou, China; 4 Department of General Surgery, The First Hospital of Lanzhou University, Lanzhou, Gansu, China; 5 State Key Laboratory of Applied Organic Chemistry, College of Chemistry and Chemical Engineering, Lanzhou University, Lanzhou, China

**Keywords:** apoptosis, cardiotoxicity, cholangiocarcinoma, lanatoside C, mitochondrial membrane potential

## Abstract

**Objective:**

Lanatoside C (Lan C), a cardiac glycoside, has demonstrated potent anti-cholangiocarcinoma activity. However, its potential cardiotoxicity at therapeutic doses remains poorly understood. This study aimed to systematically evaluate the cardiotoxicity of Lan C at cellular and organ levels and to elucidate its underlying molecular mechanisms.

**Methods:**

Human AC16 cardiomyocytes (ATCC® CRL-3568™) were employed for *in vitro* studies, with untreated cells included as negative controls. Cytotoxic effects at anti-cholangiocarcinoma-relevant concentrations (50–200 nM) were evaluated using a combination of cell viability assays, transmission electron microscopy, flow cytometry for apoptosis and reactive oxygen species (ROS) detection, JC-1 staining to assess mitochondrial membrane potential, lactate dehydrogenase (LDH) release assays, and Western blot analysis of apoptotic proteins. Male *BALB/c* mice were administered either vehicle (PBS) or Lan C (80 mg/kg/day) by oral gavage for 28 consecutive days. Cardiac function was assessed by echocardiography, and myocardial architecture was examined through histopathological analysis.

**Results:**

Lan C showed a dose-dependent inhibitory effect *in vitro* on AC16 cell viability and induced apoptosis. This process involved decreased mitochondrial membrane potential, increased reactive oxygen species (ROS), downregulation of the anti-apoptotic protein Bcl-2, and upregulation of cytochrome C, Cleaved Caspase-9, and Cleaved Caspase-3, indicating activation of the mitochondrial-dependent apoptotic pathway. *In vivo*, high-dose Lan C significantly impaired left ventricular systolic function, as indicated by reductions in LVEF and LVFS, and induced myocardial structural alterations.

**Conclusion:**

Lan C exhibits measurable but relatively low cardiotoxicity at concentrations effective against cholangiocarcinoma, with cardiac effects may be manageable at therapeutic doses. These findings support Lan C as a promising anti-tumor candidate with controllable cardiotoxicity under optimized dosing and clinical monitoring.

## Introduction

Lanatoside C (Lan C) is a cardiac glycoside belonging to the same class as digoxin and ouabain, which have been widely used to treat heart failure and arrhythmias for more than two centuries (Khandelwal et al., 2024; Slingerland et al., 2013). Lan C exerts its therapeutic effects primarily by inhibiting Na^+^/K^+^-ATPase (NKA). This inhibition increases intracellular Ca^2+^ via the Na^+^/Ca^2+^ exchanger, thereby elevating Ca^2+^ levels and eliciting an inotropic response (Chen et al., 2025; Babula et al., 2013).

Recent evidence indicates that cardiac glycosides, including Lan C, possess broad anticancer activity. These effects involve multiple mechanisms, such as disruption of intracellular ion homeostasis (Na^+^, K^+^, Ca^2+^) (Babula et al., 2013), inhibition of DNA repair and protein synthesis (Prassas and Diamandis, 2008; Surovtseva et al., 2016), induction of reactive oxygen species (ROS) (Rocha et al., 2014), modulation of tumor energy metabolism (Babula et al., 2013), alteration of biomembrane stability (Babula et al., 2013), and regulation of the tumor immune microenvironment (Zhou et al., 2025). Our previous studies demonstrated that Lan C suppresses cholangiocarcinoma cell proliferation, migration, and invasion via a mitochondrial-dependent apoptotic pathway, which includes increased ROS production, reduced mitochondrial membrane potential, and downregulation of STAT3 signaling (Zhang et al., 2023). The half-maximal inhibitory concentration (IC_50_) of Lan C in human intrahepatic bile duct epithelial cells (HIBEpiC) was substantially higher than in cancer cells, suggesting selective cytotoxicity. Moreover, long-term oral administration of Lan C at 40 mg/kg in nude mice bearing tumors did not induce significant systemic toxicity, indicating a favorable tolerability profile (Zhang et al., 2023).

Cardiac glycosides possess a narrow therapeutic window, with their efficacy and toxicity highly dose-dependent (Gonano et al., 2011; Györke and Carnes, 2008). Cardiotoxicity primarily results from intracellular calcium overload, which can precipitate myocardial injury (Finkel et al., 2015). Importantly, subclinical myocardial alterations may be reversible when the extent of damage is limited, as demonstrated by contrast-enhanced cardiac magnetic resonance studies showing functional recovery when scar transmurality is ≤ 25% (Kim et al., 2000; Pfeffer and Braunwald, 1990). Despite these observations, significant knowledge gaps persist regarding the cardiac safety profile of Lan C. Specifically, it remains unclear whether Lan C induces functionally relevant cell injury in human cardiomyocytes at therapeutically relevant concentrations, and the potential cardiac consequences of high-dose *in vivo* administration have yet to be systematically investigated.

Accordingly, this study aimed to systematically evaluate the cardiotoxicity of Lan C at both cellular and organ levels and to elucidate the underlying mechanisms. Specifically, we sought to (i) characterize its cytotoxic effects on human cardiomyocytes at concentrations relevant to cholangiocarcinoma therapy, (ii) determine the nature and extent of cardiac injury, and (iii) assess the effects of high-dose, long-term Lan C administration on cardiac structure and function *in vivo*. By addressing these aims, the study establishes a foundation for assessing the cardiac safety of Lan C and informs its potential development as a therapeutic agent for cholangiocarcinoma with minimized cardiotoxic risk.

## Materials and methods

### Cell culture and drug treatment

Human AC16 cardiomyocytes (ATCC® CRL-3568™) were cultured in DMEM supplemented with 10% FBS at 37 °C and 5% CO_2_. Lanatoside C (Lan C) was prepared in vehicle solution (PBS). Based on prior studies and preliminary dose-response assays, four concentrations (0, 50, 100, 200 nM) were selected for experiments.

### Cell viability assay

Cell viability was assessed using the Cell Counting Kit-8 (CCK-8; Dojindo, Japan). Briefly, cells were seeded in 96-well plates and cultured for 24 h, followed by treatment with Lan C at concentrations of 0, 50, 100, 250, 500, 1,000, or 2000 nM. Cells were incubated for 12, 24, or 48 h, after which CCK-8 reagent was added. Absorbance at 450 nm was measured using a microplate reader (Thermo Multiscan MK3, United States) to determine cell viability.

### Transmission electron microscopy (TEM)

Normal AC16 cells served as the control group, while experimental cells were treated with Lanatoside C for 48 h. Following treatment, cells from both groups were trypsinized, and the resulting cell pellets were collected by centrifugation. The pellets were fixed in ice-cold 2.5% glutaraldehyde in 0.1 M phosphate buffer (pH 7.4) overnight at 4 °C. Samples were subsequently post-fixed with 1% osmium tetroxide, dehydrated through a graded ethanol–acetone series, and infiltrated with epoxy resin. Resin-embedded samples were sectioned into ultrathin slices, which were double-stained with uranyl acetate and lead citrate before examination by transmission electron microscopy (TEM) (Wei et al., 2020).

### Reactive oxygen species (ROS) detection

Reactive oxygen species (ROS) levels were assessed using the fluorescent probe DCFH-DA (Elabscience, Wuhan, China). AC16 cells were seeded in 6-well plates and cultured for 24 h in complete medium. Cells were divided into a control group (untreated AC16 cells) and experimental groups treated with 50, 100, or 200 nM Lan C for 48 h. Following treatment, cells were incubated with DCFH-DA at 37 °C in the dark for 30 min. Excess probe was removed by washing with phosphate-buffered saline (PBS), and fluorescence images were acquired using a confocal microscope (LSM900, ZEISS, Germany). The mean fluorescence intensity of ROS was quantified by flow cytometry (Agilent, Palo Alto, CA, United States) (Rasheduzzaman et al., 2019).

### Mitochondrial membrane potential (ΔΨm) assay

AC16 cells were seeded in 6-well plates and cultured for 24 h in complete medium. Cells were assigned to a control group (untreated AC16 cells) or experimental groups treated with 50, 100, or 200 nM Lan C for 48 h. Following treatment, 500 μL of JC-1 staining working solution (Beyotime, Shanghai, China) was added to each well, and cells were incubated at 37 °C in the dark for 20 min. After incubation, cells were washed twice with JC-1 staining buffer and resuspended in 1 mL of DMEM. Fluorescence images were acquired using a confocal microscope (LSM900, ZEISS, Germany), and JC-1 fluorescence intensity was quantified by flow cytometry (Agilent, Palo Alto, CA, United States) (Jiang et al., 2023).

### Apoptosis assay

Apoptosis was evaluated using an Annexin V-FITC/propidium iodide (PI) detection kit (Elabscience, Wuhan, China). AC16 cells were assigned to a control group (untreated cells) or experimental groups treated with 50, 100, or 200 nM Lan C for 48 h. Following treatment, cells were collected, washed, and resuspended in 500 μL of binding buffer, then stained with 5 μL each of Annexin V-FITC and PI for 15 min in the dark. Samples were analyzed by flow cytometry (Agilent, Palo Alto, CA, United States), acquiring data from 30,000 cells per group. Early apoptotic cells were defined as Annexin V–positive/PI–negative, whereas late apoptotic cells were identified as double-positive for Annexin V and PI (Hu et al., 2018).

### Lactate dehydrogenase (LDH) release

Lactate dehydrogenase (LDH) release was measured using a Cytotoxicity Detection Kit (Elabscience, Wuhan, China). AC16 cells were seeded in 96-well plates at a density of 4,000 cells per well and cultured for 48 h in serum-free medium containing various concentrations of Lan C (0, 50, 100, or 200 nM). After incubation, 50 μL of supernatant from each well was transferred to a new 96-well plate, and an equal volume (50 μL) of assay working solution was added. The plate was gently shaken to ensure thorough mixing and incubated in the dark for 10 min. The reaction was terminated by adding the stop solution, and absorbance was measured at 450 nm with a reference wavelength above 600 nm for dual-wavelength detection. LDH activity was calculated according to the formula provided by the manufacturer (Zhang et al., 2021).

### Western blot

AC16 cells treated with various concentrations of Lanatoside C (Lan C) (0, 50, 100, 200 nM) for 48 h were harvested, and total protein was extracted using lysis buffer. The lysates were centrifuged at 12,000 × g for 10 min at 4 °C, and the supernatants were collected. Protein concentrations were determined using a BCA protein assay kit (Beyotime, Shanghai, China). Equal amounts of protein were separated by SDS-PAGE and transferred onto polyvinylidene difluoride (PVDF) membranes. Membranes were blocked with 5% non-fat milk in Tris-buffered saline containing 0.1% Tween 20 (TBST) for 2 h at room temperature and then incubated overnight at 4 °C with primary antibodies against Bcl-2 (1:5000; Huabio, China), cleaved caspase-3 (1:1000; Huabio, Hangzhou), cleaved caspase-9 (1:1000; Cell Signaling Technology, United States), cytochrome c (1:1000; Huabio, Hangzhou), and GAPDH (1:10,000; Proteintech, United States). After three washes with TBST, membranes were incubated with an HRP-conjugated goat anti-rabbit secondary antibody (1:3000; CST, United States) for 1 h at room temperature. Protein bands were visualized using enhanced chemiluminescence (ECL) reagents, imaged with a Bio-Rad ChemiDoc MP Imaging System, and quantified using ImageJ software. Protein expression levels were normalized to GAPDH as an internal control.

### Animals and experimental design

The animal experiments were approved by the Ethics Committee of the First Hospital of Lanzhou University. Male *BALB/c* mice (6 weeks old) were acclimated for 1 week under standard conditions (controlled temperature, 12 h light–dark cycle, *ad libitum* access to chow and water). Mice were randomly assigned to two groups (n = 5 per group): a control group receiving 100 μL phosphate-buffered saline (PBS) and a Lan C group receiving 80 mg/kg/day in 100 μL PBS via daily oral gavage. DMSO concentrations were matched between groups. Treatments continued for 28 consecutive days. On Day 29, cardiac function was assessed by echocardiography, after which mice were euthanized under sodium pentobarbital anesthesia, and blood and heart tissues were collected for subsequent analyses.

### Echocardiography

Cardiac function in lightly anesthetized mice was assessed using a high-resolution ultrasound system (Vevo 3100 LT, VisualSonics, Canada). Parameters measured included left ventricular ejection fraction (LVEF), fractional shortening (LVFS), end-systolic diameter (LVESD), and end-diastolic diameter (LVEDD). Control mice received PBS; Lan C-treated mice were experimental subjects. Measurements were performed using the system software.

### Heart tissue histopathology

Hearts were perfused, fixed in 4% paraformaldehyde, embedded in paraffin, and sectioned at 5 μm. Sections were stained with hematoxylin-eosin (H&E) and examined under an upright microscope. Control hearts were untreated; Lan C group hearts were experimental. Histopathological evaluation focused on myocardial fiber alignment, cytoplasmic vacuolization, and nuclear morphology (Botelho et al., 2020).

### Statistical analysis

All *in vitro* experiments were performed in triplicate and independently repeated, except for Western blotting, which was repeated five times. Continuous data are expressed as the mean ± standard deviation (SD). Comparisons between two groups were performed using Student’s t-test, while comparisons among three or more groups were conducted using one-way analysis of variance (one-way ANOVA). Statistical analyses were carried out using GraphPad Prism software (version 8.0), and a p-value <0.05 was considered statistically significant.

## Results

### Comparison of the IC_50_ of Lan C in cholangiocarcinoma cells and cardiomyocytes and selection of working concentrations

**FIGURE 1:**
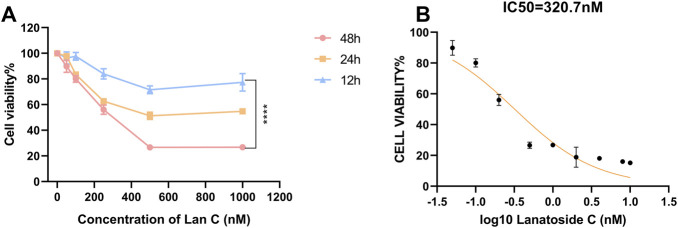
Impact of Lan C on cell viability of AC16 cells evaluated by CCK-8 assay. **(A)** Cell viability of AC16 cells treated with the indicated concentrations of Lan C for 12, 24, and 48 h. **(B)** Dose-response curve of cell inhibition on AC16 cell proliferation after 48-h treatment. The half maximal inhibitory concentration (IC50) is presented. ****P < 0.0001.

To evaluate the effect of Lan C on cardiomyocyte viability, AC16 cells were treated with different concentrations of Lan C for 12, 24, and 48 h, and cell viability was measured using the CCK-8 assay (Figure 1). AC16 cell viability decreased in a concentration- and time-dependent manner, with the strongest inhibitory effect observed after 48 h of exposure. Based on the dose–response curve generated using GraphPad Prism, the IC_50_ of Lan C in AC16 cells at 48 h was calculated to be 320 nM.

In our previous study, Lan C inhibited the proliferation of cholangiocarcinoma TFK-1 cells with an IC_50_ of approximately 103 nM at 48 h (Zhang et al., 2023). These findings indicate that AC16 cardiomyocytes were less sensitive to Lan C than cholangiocarcinoma cells under the same exposure conditions. Therefore, concentrations of 0, 50, 100, and 200 nM were selected for subsequent *in vitro* experiments, as these concentrations fall within the effective anti-cholangiocarcinoma range while remaining below the IC_50_ in AC16 cells.

### Lan C reduces AC16 cell viability and induces apoptotic and ultrastructural changes

**FIGURE 2:**
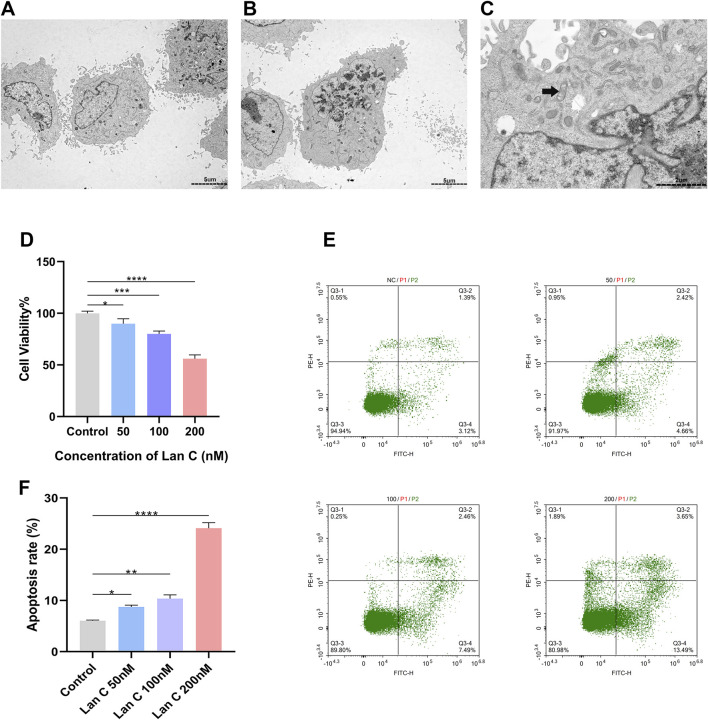
Transmission electron microscopy (TEM), cell viability, and flow cytometry of apoptosis following 48-h treatment with anti-cholangiocarcinoma concentrations of Lan C. The ultrastructure of cell samples was observed by TEM at 2000× **(A,B)** and 7000× magnifications **(C)**; **(A)** Control group; **(B)** 200 nM Lan C group: The nucleus was deformed and pyknotic, with highly condensed chromatin **(C)** 200 nM Lan C group: Damaged mitochondria with indistinct cristae were observed; **(D)** Changes in cell viability rate after 48-h treatment with different concentrations of Lan C; **(E)** Flow cytometry plots of apoptosis after 48-h treatment with different concentrations of Lan C. **(F)** Flow cytometry statistics of cell apoptosis after 48 h of treatment with different concentrations of Lan C. *P < 0.05; **P < 0.005; ***P < 0.0005; ****P < 0.0001. Scale bars: 5 μm and 2 μm.

To further characterize the effects of Lan C on AC16 cells, ultrastructural changes were examined by transmission electron microscopy. In the control group, AC16 cells showed a typical spindle-shaped morphology, regular nuclei, mitochondria with intact cristae, and normal endoplasmic reticulum (Figure 2A). In contrast, AC16 cells treated with 200 nM Lan C exhibited marked ultrastructural abnormalities, including mitochondrial swelling, disorganized or absent cristae, nuclear deformation, and chromatin condensation (Figures 2B,C). These findings indicate that Lan C induces both organelle injury and apoptotic morphological changes in AC16 cells.

Consistent with these observations, Lan C reduced AC16 cell viability in a concentration-dependent manner. After 48 h of treatment, cell viability decreased by approximately 10%, 20%, and 44% in the 50, 100, and 200 nM groups, respectively, compared with the control group (Figure 2D). Annexin V/PI flow cytometry further showed a concentration-dependent increase in apoptosis. The apoptotic rate was 6.033% ± 0.1185% in the control group, 8.737% ± 0.3612% in the 50 nM group, 10.37% ± 0.7220% in the 100 nM group, and 24.11% ± 1.837% in the 200 nM group (Figures 2E,F). Together, these data demonstrate that Lan C decreases viability and promotes apoptosis in AC16 cells within the concentration range relevant to its anti-cholangiocarcinoma activity.

### Lan C induces mitochondrial dysfunction in AC16 cells

**FIGURE 3:**
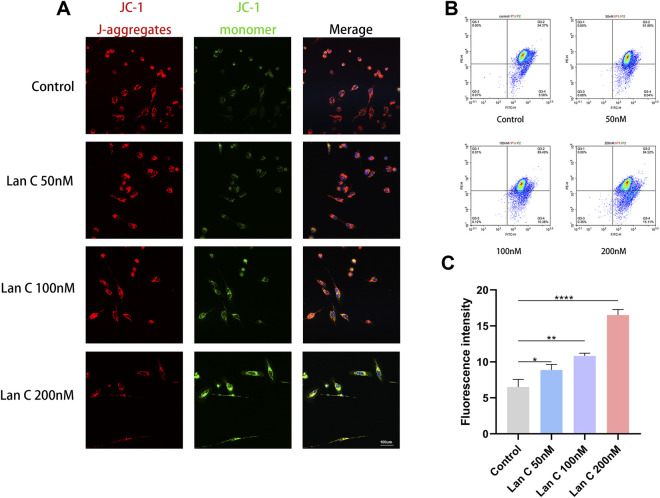
Effects of Lan C on Mitochondrial Membrane Potential Assessed by Confocal Microscopy and Flow Cytometry. **(A)** Representative confocal microscopy images of JC-1 staining. **(B)** Representative flow cytometry density plots of JC-1 staining. **(C)** Quantitative analysis of JC-1 monomer (green fluorescence) intensity by flow cytometry. Data are presented as mean ± SD. *P < 0.05, **P < 0.005, ****P < 0.0001. Scale bars: 100 μm.

Because mitochondrial dysfunction is closely associated with apoptosis, mitochondrial membrane potential was assessed using the JC-1 probe. After 48 h of treatment with 50, 100, or 200 nM Lan C, confocal microscopy showed a progressive decrease in red fluorescence intensity and a corresponding increase in green fluorescence intensity, indicating a concentration-dependent loss of mitochondrial membrane potential (Figure 3A).

Flow cytometric analysis further confirmed this result. The mean fluorescence intensity of JC-1 monomers was 6.513 ± 1.07 in the control group and increased to 8.873 ± 0.77, 10.82 ± 0.38, and 16.5 ± 1.35 in the 50, 100, and 200 nM groups, respectively. All Lan C-treated groups differed significantly from the control group (Figures 3B,C). These findings indicate that Lan C induces mitochondrial depolarization in AC16 cells in a dose-dependent manner.

### Lan C increases intracellular ROS levels in AC16 cells

**FIGURE 4:**
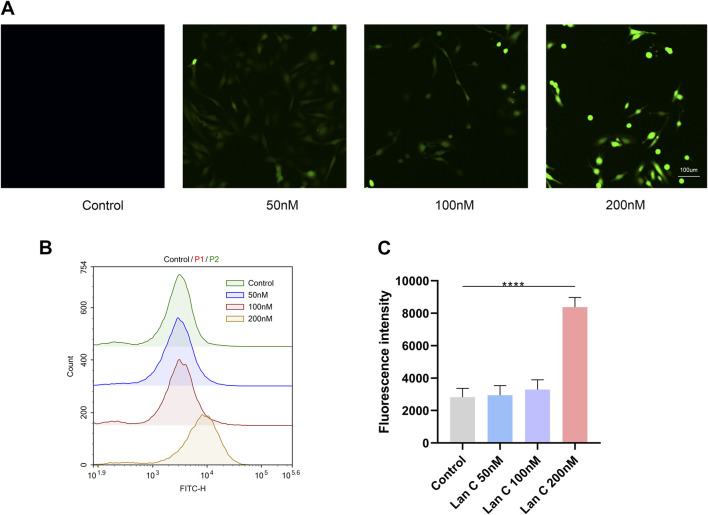
Effects of Lan C on Cell Viability and Reactive Oxygen Species (ROS) Levels in AC16 Cells. **(A)** Representative confocal microscopy images showing ROS-associated fluorescence intensity in cells treated with the indicated concentrations of Lan C for 48 h. **(B)** ROS fluorescence intensity was measured by flow cytometry in cells treated with the indicated concentrations of Lan C for 48 h. **(C)** Quantitative analysis of ROS fluorescence intensity obtained from flow cytometry. Data are presented as mean ± SD. **P < 0.005, ****P < 0.0001. Scale bars: 100 μm.

To determine whether oxidative stress was involved in Lan C-induced injury, intracellular ROS levels were measured using the DCFH-DA fluorescent probe. Confocal microscopy showed that green fluorescence intensity increased progressively with increasing Lan C concentrations, indicating enhanced intracellular ROS accumulation (Figure 4A).

This finding was supported by flow cytometric analysis, which showed that ROS production increased in a concentration-dependent manner after 48 h of treatment with 0, 50, 100, and 200 nM Lan C (Figure 4B). A statistically significant increase was observed in the high-concentration group compared with the control group (Figure 4C). These results indicate that Lan C promotes oxidative stress in AC16 cells and suggest that ROS accumulation may contribute to the observed mitochondrial dysfunction and apoptosis.

### Lan C alters apoptosis-related protein expression and increases membrane damage in AC16 cells

**FIGURE 5:**
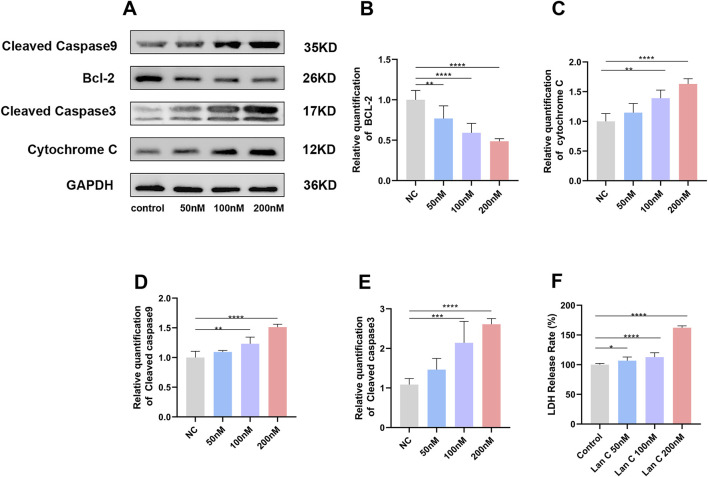
Analysis of mitochondrial-mediated apoptotic pathway protein expression and lactate dehydrogenase (LDH) release in AC16 cells following Lan C treatment. **(A)** Representative Western blot bands showing protein expression levels of Bcl-2, cytochrome c, cleaved caspase-9, and cleaved caspase-3. **(B–E)** Quantitative analysis of the relative band intensity (gray value) for Bcl-2 **(B)**, cytochrome c **(C)**, cleaved caspase-9 **(D)**, and cleaved caspase-3 **(E)**. **(F)** The release rate of LDH into the extracellular medium after AC16 cells were treated with Lan C. *P< 0.05; **P < 0.005; ****P < 0.0001.

To further investigate the mechanism underlying Lan C-induced apoptosis, the expression of key mitochondrial apoptosis-related proteins was analyzed by Western blotting. The anti-apoptotic protein Bcl-2 was downregulated in a concentration-dependent manner after Lan C treatment, and this reduction was significant in all treated groups compared with the control group. The decrease was more pronounced in the 100 and 200 nM groups (Figures 5A,B).

By contrast, the expression levels of cytochrome c, cleaved caspase-9, and cleaved caspase-3 increased with increasing Lan C concentration. Significant upregulation of these proteins was observed in the 100 and 200 nM groups, whereas no significant difference was detected in the 50 nM group relative to the control group (Figures 5A,C–E). These changes were consistent with the apoptosis data obtained by flow cytometry, supporting the involvement of the mitochondrial apoptotic pathway in Lan C-induced AC16 cell death.

To assess whether Lan C also affected plasma membrane integrity, LDH release into the culture supernatant was measured. After 48 h of treatment with 50–200 nM Lan C, LDH activity was significantly increased compared with the control group, and the highest level was detected in the 200 nM group (Figure 5F). The increase in LDH release occurred in a concentration-dependent manner, indicating that Lan C not only induces apoptosis but also compromises membrane integrity in AC16 cells.

### Long-term administration of high-dose Lan C impairs cardiac function and myocardial structure in mice

**FIGURE 6:**
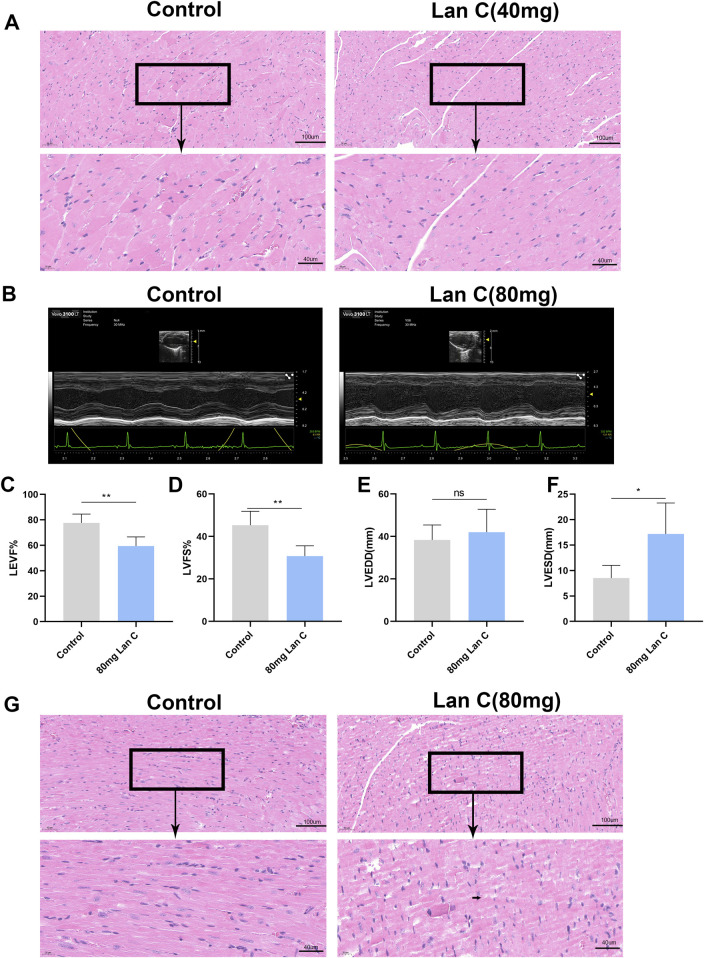
Lan C-induced cardiac functional impairment and histopathological alterations *in vivo*. **(A)** Representative hematoxylin and eosin (H&E)-stained images (20× and 40×) of heart tissue from tumor-bearing mice in a prior study, showing results from the control and 40 mg/kg Lan C-treated groups. **(B)** Representative echocardiograms from the present study, comparing the control group with the 80 mg/kg Lan C-treated group. **(C–F)** Quantitative analysis of cardiac functional parameters **(C)** Left ventricular ejection fraction (LVEF), **(D)** Left ventricular fractional shortening (LVFS), **(E)** Left ventricular end-diastolic diameter (LVEDD), and **(F)** Left ventricular end-systolic diameter (LVESD). **(G)** H&E-stained sections of myocardial tissue (control and 80 mg/kg Lan C-treated groups). Arrows indicate typical degenerative vacuolar structures. *P < 0.05; **P < 0.005. Scale bars: 50 μm and 20 μm.

In our previous study, oral administration of Lan C at 40 mg/kg significantly reduced tumor volume and weight in nude mice bearing subcutaneous tumors and did not cause obvious cardiopathological abnormalities on heart histology (Figure 6A). In the present study, to further evaluate the cardiac effects of Lan C *in vivo*, the dose was increased to 80 mg/kg, and *BALB/c* mice received oral gavage for 28 consecutive days.

Echocardiographic analysis showed that high-dose Lan C significantly impaired left ventricular systolic function. Compared with the control group, Lan C-treated mice exhibited lower left ventricular ejection fraction (LVEF; 80.84% ± 8.163% vs. 63.02% ± 10.22%) and left ventricular fractional shortening (LVFS; 49.96% ± 11.31% vs. 33.62% ± 7.609%). In parallel, left ventricular end-diastolic diameter and left ventricular end-systolic diameter were significantly increased (Figures 6B–F).

Histopathological analysis further supported these findings. In the control group, myocardial fibers were regularly arranged, nuclei appeared normal, and the interstitial structure was intact. In contrast, the Lan C-treated group showed disorganization of myocardial fibers and cytoplasmic vacuolization in cardiomyocytes (Figure 6G). Collectively, these results demonstrate that long-term administration of high-dose Lan C induces functional and structural cardiac injury *in vivo*.

## Discussion

The repurposing of cardiac glycosides for anticancer therapy has attracted increasing interest in recent years. Among these compounds, Lan C has shown promising antitumor activity in cholangiocarcinoma and other malignancies, but its potential cardiac toxicity remains a major concern for further translational development (Kumavath et al., 2021; Johansson et al., 2001). As a member of the cardiac glycoside family, Lan C is expected to have a narrow therapeutic window, and its toxic effects are likely to be strongly dependent on exposure level (Mijatovic et al., 2007). Therefore, a key issue in evaluating its translational value is whether antitumor efficacy can be achieved within an exposure range that does not produce unacceptable cardiotoxicity.

In the present study, we systematically evaluated the cardiotoxic effects of Lan C at concentrations relevant to its anti-cholangiocarcinoma activity and further assessed the consequences of higher-dose exposure *in vivo*. One of the most important findings of this study is that the toxic response of cardiomyocytes to Lan C was clearly concentration-dependent. The IC_50_ of Lan C in AC16 cardiomyocytes was 320 nM, whereas the IC_50_ previously observed in TFK-1 cholangiocarcinoma cells was approximately 103 nM(Zhang et al., 2023), indicating an approximately 3.1-fold difference in sensitivity under the same exposure duration. This quantitative comparison suggests that Lan C retains a certain degree of selectivity toward tumor cells, although the safety margin appears modest rather than wide. More importantly, within the concentration range of 50–200 nM, which overlaps with its effective anti-cholangiocarcinoma range, AC16 cell viability declined progressively by approximately 10%, 20%, and 44%, while the apoptotic rate increased from 6.03% in control cells to 8.74%, 10.37%, and 24.11%, respectively. These data indicate that cardiomyocyte injury was not an all-or-none phenomenon, but instead became progressively more pronounced as Lan C concentration increased. This concentration-dependent pattern is particularly important because it suggests that even within the therapeutically relevant range, cardiotoxic effects may intensify substantially as exposure rises.

This concentration-dependent phenomenon was also consistently reflected at the subcellular and molecular levels. As a key indicator of cellular health, mitochondrial functional integrity is typically assessed by mitochondrial membrane potential (MMP) (Demine et al., 2019). Maintenance of MMP is essential for normal mitochondrial function, and its decline often represents a critical early event in apoptosis (Wen et al., 2015). In this study, JC-1 staining results demonstrated that Lan C concentration-dependently reduced MMP in AC16 cells, suggesting opening of the mitochondrial permeability transition pore (mPTP) and initiation of early apoptosis (Kang et al., 2021). Within the concentration range of 50–200 nM, which closely overlaps with its effective concentration for cholangiocarcinoma treatment, MMP was significantly decreased. As Lan C concentration increased, mitochondrial depolarization became more severe, accompanied by a corresponding increase in the apoptotic rate.

Oxidative stress plays an important role in cardiac glycoside-induced cardiotoxicity (Luczak and Anderson, 2014). The production of ROS in cardiac injury induced by cardiac glycosides mainly stems from two aspects. On one hand, intracellular calcium overload is taken up by the mitochondrial calcium uniporter, leading to mitochondrial calcium overload, which in turn disrupts the electron transport chain and triggers a burst of ROS within the mitochondria (Carvalho et al., 2020; Palma et al., 2024). On the other hand, the binding of cardiac glycosides to the Na^+^/K^+^-ATPase activates the Src kinase signaling pathway, which subsequently activates NADPH oxidase in the plasma membrane, resulting in ROS production (Ho et al., 2014; Viatchenko-Karpinski et al., 2012). These two types of ROS promote each other and further stimulate the opening of the mPTP, ultimately initiating the apoptotic program (Crola Da Silva et al., 2023). In this study, Lan C treatment substantially elevated intracellular ROS levels in AC16 cells. Although ROS elevation showed a concentration-dependent trend, a statistically significant increase was observed only in the group treated with 200 nM Lan C. This finding suggests that Lan C likely induces mitochondrial damage and apoptosis by significantly exacerbating oxidative stress at relatively high concentrations.

Meanwhile, apoptosis-related alterations became increasingly pronounced at higher concentrations, as evidenced by reduced Bcl-2 expression and increased levels of cytochrome c, cleaved caspase-9, and cleaved caspase-3. Given that Bcl-2 is a key mitochondrial anti-apoptotic protein responsible for preserving membrane integrity and limiting cytochrome c release (Czabotar et al., 2014), its downregulation suggests a loss of mitochondrial membrane stability, which may in turn facilitate cytochrome c release and trigger the caspase-dependent apoptotic cascade (Xu et al., 2024). Consistent with this interpretation, the increased release of LDH, a cytosolic marker of membrane damage, further indicates that cellular injury became progressively more severe with increasing exposure (Timmins et al., 2024). Taken together, these findings support a concentration-dependent pattern of Lan C-induced cardiotoxicity, characterized by progression from early mitochondrial dysfunction and oxidative stress to apoptotic activation and more extensive structural injury. This pattern further implies that mitochondrial impairment and redox imbalance are early mechanistic events in Lan C-induced toxicity. Accordingly, dose dependence should be regarded as a defining feature of its cardiac safety profile, and careful control of exposure may be critical for limiting mitochondrial injury and excessive ROS accumulation within its therapeutic window.

This interpretation should be viewed alongside previous evidence suggesting that Lan C may exhibit a relatively acceptable acute toxicity profile compared with other cardiac glycosides. In a guinea pig model, Rand and Staffer reported a median lethal dose of 0.679 mg/kg for Lan C, which exceeded that of ouabain (0.232 mg/kg) and digoxin (0.588 mg/kg), thereby indicating a comparatively more favorable acute toxicity profile (Rand and Stafford, 1959). In addition, its rapid onset of action, short duration of effect (approximately 37 min), and high elimination rate (295.7 μg/kg/h) may further contribute to its relative safety under acute exposure conditions (Rand and Stafford, 1959). Previous studies have also shown that orally administered Lan C must undergo partial metabolism to digoxin before absorption and that its higher molecular weight (985) may result in approximately 20% lower systemic exposure than milligram-equivalent doses of digoxin (Aldous and Thomas, 1977). This pharmacokinetic feature may, at least in part, reduce the likelihood of drug accumulation. Moreover, recent *in vitro* studies have demonstrated that Lan C exerts cytotoxic effects in several tumor cell lines, including A549, HeLa, and MCF-7 cells, with IC_50_ values ranging from 0.13 to 0.24 µM, and may display a selectivity index greater than 1.5 relative to certain normal cells (Yang et al., 2022). When considered together with the present findings, these observations support the possibility that Lan C may possess an exploitable therapeutic window. However, our data also indicate that this window is likely narrow, as cardiomyocyte injury increased progressively when concentrations approached or exceeded the lower end of the reported antitumor-effective range. Therefore, the therapeutic potential of Lan C lies not in the absence of cardiotoxicity, but rather in the possibility that systemic exposure can be controlled within a range that preserves antitumor efficacy while maintaining cardiac toxicity at a comparatively limited level.

In our previous tumor-bearing mouse study, long-term administration of 40 mg/kg Lan C did not produce overt cardiac histopathological abnormalities (Zhang et al., 2023), whereas in the present study, 80 mg/kg administered for 28 consecutive days caused clear impairment of left ventricular systolic function and histopathological myocardial injury. The contrast between these two regimens indicates that relatively modest increases in dose may shift Lan C from a comparatively tolerated range into a clearly cardiotoxic range. This observation has direct translational relevance, as it implies that the safety of Lan C is highly sensitive to exposure escalation and that the upper boundary of its usable dose range may be narrow (Zhai et al., 2022).

From a clinical and translational perspective, these findings further clarify how the potential safety window of Lan C should be interpreted. Our data suggest Lan C may retain antitumor activity at certain concentrations. At these concentrations, cardiomyocytes appear less sensitive than cholangiocarcinoma cells. However, this separation is only partial and becomes progressively narrower as exposure increases. Thus, the present study does not imply that therapeutically relevant concentrations are devoid of cardiac risk. Rather, it suggests that cardiac injury may remain comparatively limited at lower effective exposures but becomes increasingly apparent with rising concentration. This distinction is clinically relevant because it supports a development strategy centered on defining the minimum effective antitumor concentration, minimizing cumulative exposure, and implementing careful cardiac safety monitoring during treatmen (Turner et al., 2014; Oren et al., 2021).

Several limitations of this study should also be acknowledged. First, the toxic effects of cardiac glycosides differ substantially across species, and rodents are generally more tolerant than humans to this class of compounds (Senn et al., 1988). Accordingly, the safety margin observed in mice may overestimate human tolerability (Senn et al., 1988). Second, the sample size in the animal experiments was relatively small. Third, although tumor-bearing models may more closely approximate the clinical setting, they were not utilized in the present study. Future translational studies incorporating such models are warranted to better assess the cardiac safety profile of Lan C. Fourth, the present study focused primarily on structural and functional myocardial injury and did not assess electrophysiological toxicity, which represents a clinically important manifestation of cardiac glycoside–associated adverse effects and therefore warrants particular attention (Ho et al., 2011). Given the well-established proarrhythmic liability of this drug class, future studies should incorporate electrophysiological evaluation, ideally in larger cohorts and in models that more closely approximate human physiology. Such studies are needed to define more precisely the exposure range within which Lan C may preserve antitumor activity while minimizing cardiac risk.

In summary, this study demonstrates that the cardiotoxicity of Lan C is strongly concentration- and dose-dependent. Quantitative comparison showed that AC16 cardiomyocytes were less sensitive than TFK-1 cholangiocarcinoma cells to Lan C, supporting a degree of tumor selectivity, but this selectivity was limited. More importantly, as concentration increased, the cytotoxic effects of Lan C on cardiomyocytes became progressively more severe, as reflected by reduced viability, increased apoptosis, worsening mitochondrial dysfunction, enhanced ROS accumulation, activation of the mitochondrial apoptotic pathway, and increased membrane damage. *In vivo*, higher-dose and prolonged exposure similarly resulted in clear impairment of cardiac structure and function. Collectively, these findings suggest that Lan C may possess a narrow but potentially actionable therapeutic window, and that future development should focus on strict exposure control, dose optimization, and rigorous cardiac safety evaluation (Nik Nabil et al., 2024).

## Conclusion

In summary, this study systematically characterized the cardiotoxicity of Lan C at concentrations relevant to its anti-cholangiocarcinoma activity. *In vitro*, Lan C induced concentration-dependent injury in human cardiomyocytes, as evidenced by increased ROS accumulation, loss of mitochondrial membrane potential, activation of the mitochondrial apoptotic pathway, and membrane damage. *In vivo*, prolonged exposure to a higher dose impaired left ventricular systolic function and induced myocardial structural injury in mice, indicating that its cardiotoxicity is also dose-dependent at the organismal level.

Together with our previous findings of antitumor efficacy and relatively acceptable tolerability at lower exposure levels, these results suggest that Lan C may have a narrow but potentially actionable therapeutic window. However, this window appears to be strongly exposure-dependent, and the separation between antitumor activity and cardiotoxicity is likely limited. These findings provide a basis for future studies on dose optimization, exposure control, and the development of Lan C derivatives with reduced cardiotoxicity.

## Data Availability

The original contributions presented in the study are included in the article/Supplementary Material, further inquiries can be directed to the corresponding authors.
