# Supplementary material for: Evaluation and investigation of the cardiotoxicity of the potential anti-cholangiocarcinoma drug lanatoside C
Source: Front Toxicol. 2026 May 25;8:1783444. doi: 10.3389/ftox.2026.1783444 (PMC13242897; doi:10.3389/ftox.2026.1783444)
Supplement: Supplementary file 1 [file DataSheet1.pdf]

Regarding the Post Power calculations Analysis for Animal Experiments

LVEF

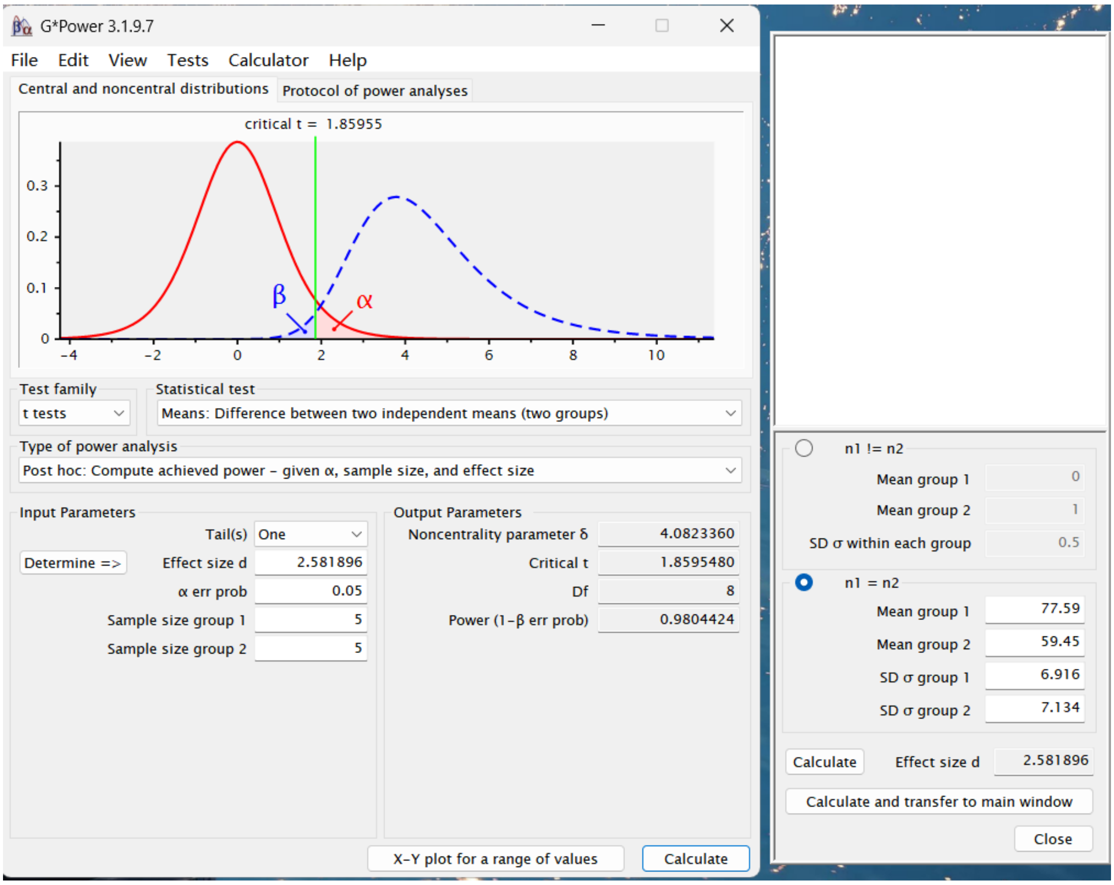

LVFS

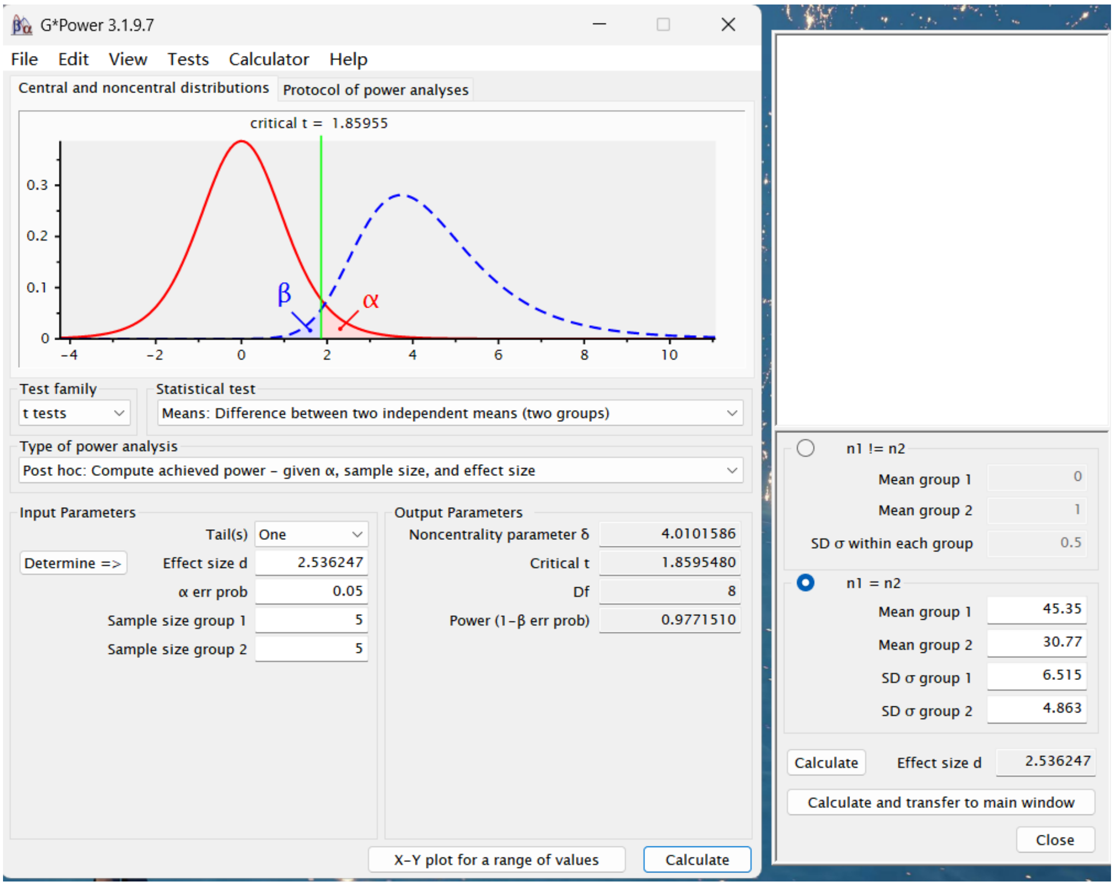

LVEDD: There was no difference in either the article or the experiment.

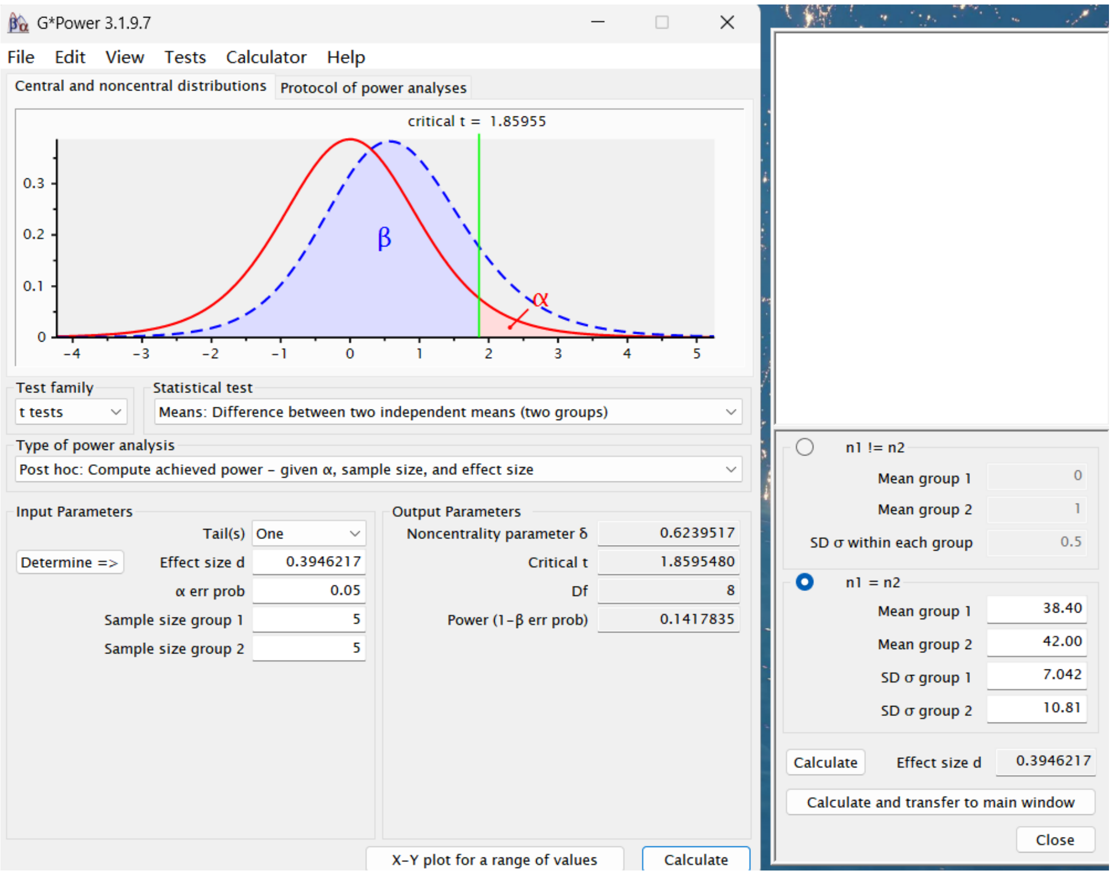

LVESD

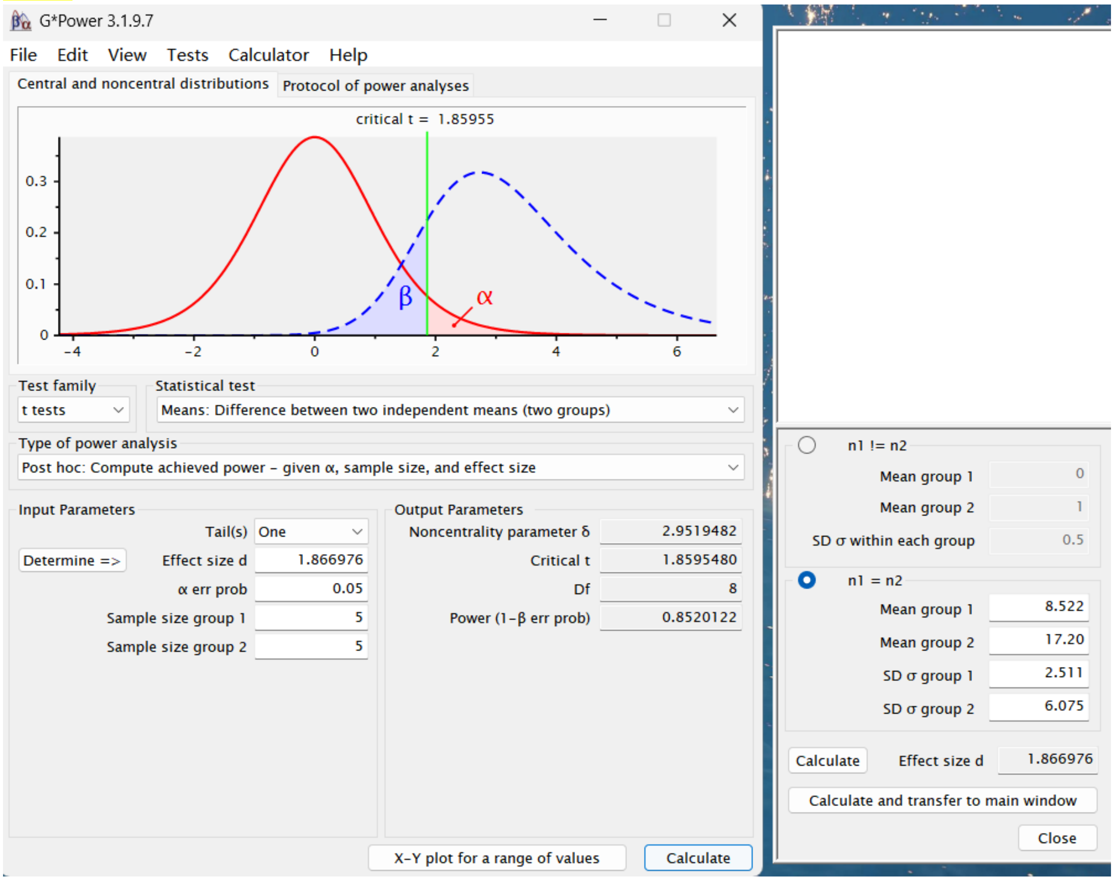
G*Power 3.1.9.7
File Edit View Tests Calculator Help
Central and noncentral distributions
Protocol of power analyses
critical t = 1.85955
Test family
t tests
Statistical test
Means: Difference between two independent means (two groups)
Type of power analysis
Post hoc: Compute achieved power – given α, sample size, and effect size
Input Parameters
Tail(s) One
Determine =>
Effect size d 1.866976
α err prob 0.05
Sample size group 1 5
Sample size group 2 5
Output Parameters
Noncentrality parameter δ 2.9519482
Critical t 1.8595480
Df 8
Power (1−β err prob) 0.8520122
X-Y plot for a range of values
Calculate
n1 != n2
Mean group 1 0
Mean group 2 1
SD σ within each group 0.5
n1 = n2
Mean group 1 8.522
Mean group 2 17.20
SD σ group 1 2.511
SD σ group 2 6.075
Calculate
Effect size d 1.866976
Calculate and transfer to main window
Close
